# Evaluation of allogeneic freeze-dried platelet lysate in cartilage exposed to interleukin 1-β in vitro

**DOI:** 10.1186/s12917-019-2118-z

**Published:** 2019-11-01

**Authors:** Livia Camargo Garbin, C. Wayne McIlwraith, David D. Frisbie

**Affiliations:** 1 Department of Clinical Veterinary Sciences, School of Veterinary Medicine, Faculty of Medical Sciences, University of West Indies, St. Augustine Campus, St. Augustine, Trinidad and Tobago; 2 C.Wayne McIlwraith Translational Medicine Institute, Orthopaedic Research Center, Colorado State University, 2350 Gillette Drive, Fort Collins, CO 80523 USA

**Keywords:** Platelet-rich plasma, Allogeneic, Freeze-dried, Equine, Cartilage

## Abstract

**Background:**

Platelet-rich plasma (PRP) as well as other platelet-derived products have been used as a potential disease-modifying treatment for musculoskeletal diseases, such as osteoarthritis (OA). The restorative properties of such products rely mainly on the high concentrations of growth factors, demonstrating encouraging results experimentally and clinically. Yet, the autologous blood-derived nature of the PRP product lead to limitations that precludes it’s widespread use. The main limitations for PRP use are; product variability, the need for minimum laboratory settings in most cases, and the need for storage at low temperatures to preserve its properties. Based on these limitations, the objective of this study was to investigate an allogeneic off-the-shelf platelet lysate (PL) in cartilage exposed to interleukin 1β (IL-1β). For this purpose, blood and cartilage were harvested from eight skeletally mature and healthy horses. Blood was processed into PL aliquots and divided into three groups (Frozen, Freeze-dried and Filtered freeze-dried), used in autologous and allogeneic conditions and in three different concentrations (1.5, 3 and 6-fold). Different PL preparations were then applied in cartilage culture with interleukin-1 beta and cultured for 10 days. Cartilage and media samples were collected and analyzed for total GAG and ^35^SO_4_-labeled GAG content.

**Results:**

No significant differences between the controls and PL groups in cartilage and media were demonstrated. The effects of PL on cartilage matrix were concentration dependent and intermediate concentrations (3-fold) in PL showed increased ^35^SO_4_-labelled GAG in cartilage.

**Conclusion:**

In conclusion, the allogeneic freeze-dried PL presented equivalent effects compared to frozen autologous PL. Intermediate platelet concentration on average demonstrated improved results, demonstrating less GAG loss compared to other concentrations.

## Background

Osteoarthritis (OA) is one of the most important causes of equine and human musculoskeletal disability. Loss of homeostasis in favor of catabolic activities is believed to contribute to the progressive degeneration characteristic of OA [1]. Therefore, treatments that focus on the interaction of numerous mediators necessary for joint homeostasis and cartilage growth could offer a novel disease modifying option for OA. Therapies like platelet-rich plasma (PRP) or platelet lysate (PL) that deliver bioactive factors have both experimentally and anecdotally been reported to offer a potential treatment for this disease [2, 3].

Platelet-rich plasma has shown promising results in alleviating clinical signs in early OA in human patients [4]. This therapy demonstrated analgesic and anti-inflammatory properties [5] in human [6] and equine studies [7]. The clinical improvement in patients treated with PRP is believed to be explained by the action of growth factors release by activated platelets. Growth factors including transforming growth factor- β (TGF-β) and platelet-derived growth factor (PDGF) are known to modulate tissue inflammation and healing [8]. Through anabolic effects and the inhibition of metalloproteinases, platelet products may mediate and promote cartilage healing [9].

While positive clinical results encourage the use of PRP, the optimization of this treatment is still warranted. Areas of improvement include optimal preparation methods, dose, treatment timing, frequency of application and stable storage methods [10, 11]. Methods to decrease patient variability is also a target for optimization. In this study, we addressed the issues of storage, product variability and in most cases the need for special equipment for PRP preparation by testing an allogeneic freeze-dried version of platelet-derived product, referred to here as PL. Based on this objective, the central hypothesis of this study is that allogeneic freeze-dried PL will have equivalent biologic effects compared to frozen PL on cartilage stimulated with IL-1β in vitro.

## Results

### Platelet lysate creation

After the first centrifugation, the plasma above the buffy coat was collected. The platelets as well as white blood cells (WBC) in this fraction were counted. The average baseline automated platelet count was 273.25 × 10^3^ platelets/μL (202–368 platelets/μL) for the 8 horses. Manual counting resulted in an average of 291.8 × 10^3^ platelets/μL. An average value of 282.52 platelets × 10^3^ platelets/μL was used in this experiment as a baseline platelet number to calculate different concentrations of PL (1.5, 3 and 6-fold). Following centrifugation, the platelet counts of the supernatant plasma collected were within normal range for the horse (125–300 × 10^3^ platelets/μL^1^). The average nucleated cell count was 812.5 cells/μL using an automated method. This value was below the normal range of WBC count for peripheral blood of horses (5500–10,500 cells/μL).^2^ After rehydration of the pellets with media, platelet concentrations were estimated at 423 (1.5-fold), 847 (3-fold) and 1695 (6-fold) × 10^3^ platelets/μL respectively. Nucleated cell counts were estimated at 1.2 (1.5-fold), 2.4 (3-fold) and 4.8 (6-fold) × 10^3^ cells/μL respectively.

### Stimulation of explant with IL-1β

Media GAG was significantly (*P* ≤ 0.0226) increased on Days 2 & 6 in the presence of IL-1β (Day 2; ITS+IL-1β: 74.28 ± 17.03, FBS + IL-1β: 68.58 ± 17.82 and Day 6; ITS+ IL-1β: 54.95 ± 17.92 and FBS+ IL-1β: 62.32 ± 20.21 μg/μg of DNA), when compared to no IL-1β (Day 2; ITS only: 38.19 ± 17.76, FBS only: 33 ± 17.81 and Day 6; ITS only: 26.13 ± 20.12; and FBS only: 9.11 ± 20.21 μg/μg of DNA) independent of the media used (ITS or FBS). ^35^SO_4_ -labeled GAG remaining in the cartilage explants at the termination of the experiment showed less labeled GAG remaining. This suggested increased degradation in both ITS and FBS media, however such differences were not significant (P: 0.1951).

### Ability of PL to affect IL-1β stimulation

**Fig. 1:**
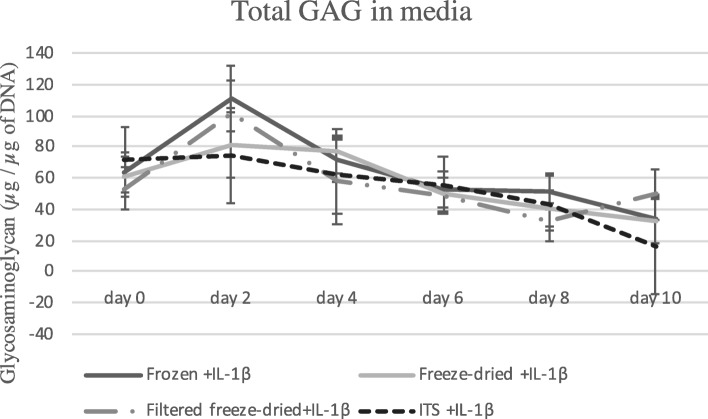
Total glycosaminoglycan (GAG) in media during the experiment. Cartilage explants were submitted to IL-1β and treated with different formulations of PL and compared to control (ITS+ILβ). No significant difference was observed for the amount of GAG released into the media during the experiment, between the cartilage samples treated with PL and the stimulated control ITS+ILβ. Level of significance 0.05

Independent of Treatment, Allogeneicity or Concentration PL did not demonstrate any statistical differences when compared to ITS in the presence of IL-1 for any outcome parameter. Glycosaminoglycan content released into the media during the experiment can be observed on Fig. 1.

### Treatment, Allogenicity and Concentration

**Fig. 2:**
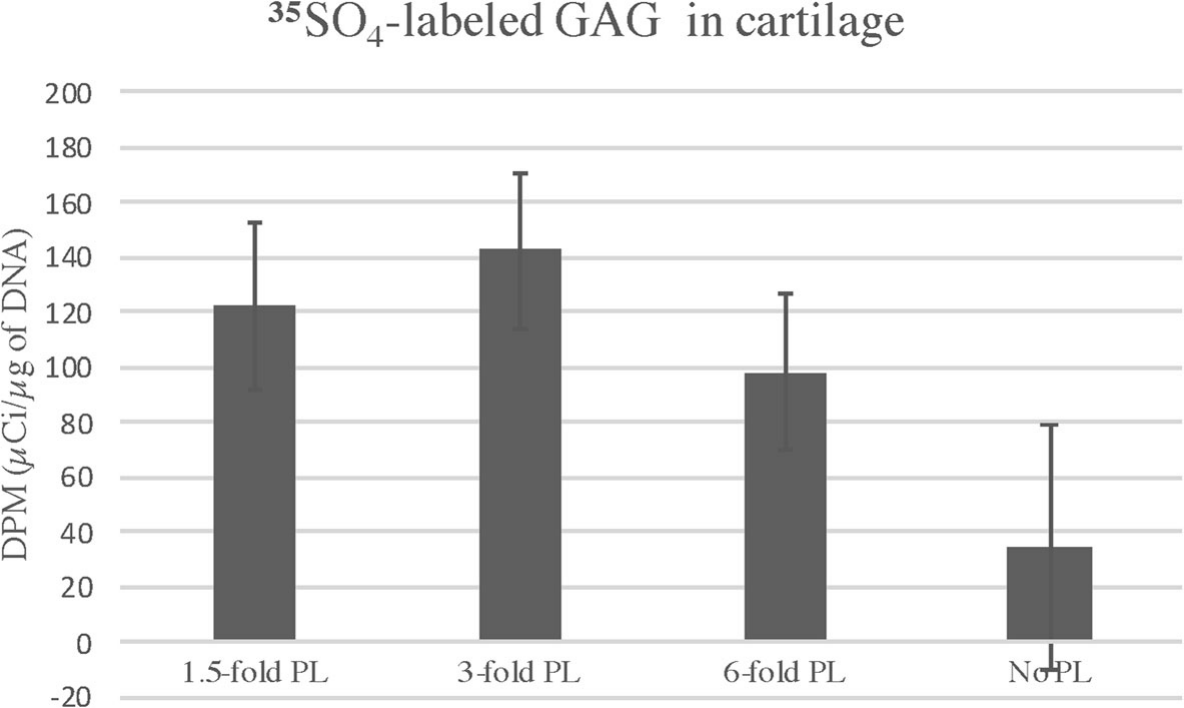
^35^SO_4_-labeled GAG in cartilage treated with different concentrations of PL. No significant effect was observed for the effect of concentration (P:0.2972). However, the ^**35**^SO_4_-labeled GAG observed in cartilage treated with PL, had numerically higher values compared to the controls, especially for the 3-fold concentration, which presented the highest ^**35**^SO_4_-labeled GAG value, but still not statistically significant when compared to ITS media control (P:0.078). The lack of statistical difference could be explained by the high variability between the horses. Values are represented as means (DPM/μg of DNA), and bars represent standard errors of the mean. Level of significance 0.05

No significant differences were observed in GAG released into the media or total GAG in cartilage explants based on any of the fixed effects or their interactions. Samples treated with 3-fold PL (114.71 ± 14.66 DPM/μg of DNA) demonstrated significantly more ^35^SO_4_- labeled GAG retained in cartilage compared to samples treated with 1.5-fold PL (60.99 ± 14.22 DPM/μg of DNA, *P* = 0.0065) or 6-fold PL (69.88 ± 14.216 DPM/μg of DNA *P* = 0.017). When individual comparisons were made, there was more labeled cartilage GAG in 3-fold concentration of the Freeze-dried treatment compared to the other concentrations. However, when controls were taken into consideration, no significant changes were observed for samples treated with different concentrations of PL compared to controls (P:0.2972. Fig. 2). In addition, no significant increase in ^35^SO_4_- labeled GAG retained in cartilage was observed for samples submitted to different PL treatments in various concentrations compared to controls (P:0.3053. Table 1).

**Table 1:** ^35^SO_4_- labeled GAG retained in cartilage by the end of the experiment. Samples were treated with different platelet derived products (PL) in different concentrations and compared to controls (using ITS or FBS as medium supplementation)

Platelet-lysate Product or control	0-fold (Control)	1.5-fold	3-fold	6-fold
Frozen PL	*******	46.5137 ± 39.2634	85.696 ± 36.1581	67.0945 ± 34.8874
Freeze-dried PL	*******	68.4964 ± 33.7408	146.34 ± 36.2139	74.4229 ± 34.8874
Filtered freeze-dried PL	*******	166.96 ± 39.2691	111.14 ± 36.2195	68.5834 ± 34.9405
ITS control	75.7266 ± 52.7041	***********	***********	***********
FBS control	79.3627 ± 48.8237	***********	***********	***********

No significant difference was observed for the main comparisons of PL*Concentration interaction (P:3053). Values in table represent mean ± standard error of the mean in DPM/ μg of DNA. Level of significance 0.05

## Discussion

This study utilized IL-1β to create an inflammatory environment to test the biological equivalency of the various PL preparations in an in vitro system. Our primary goal was to compare the biologic activity of allogeneic freeze-dried PL to frozen autologous PL. We also wanted to evaluate the effect that different concentrations of platelets may have in this system.

The baseline platelet count for the PRP used for the preparation of the different PL formulations was confirmed to be within normal range for horses.^3^ After termination of PL processing, the PL used at 1.5, 3 and 6-fold concentrations presented increased concentration of platelets compared to whole blood by definition and is equivalent or higher compared to double spin protocols tested elsewhere [11]. The WBC concentration found in all the various PL products produced, were below baseline for equine blood, and considered low compared to highly concentrated PRP [11]. While consensus is not uniform over the platelet or WBC concentration that is optimal for joint related issues, we choose a low WBC product based on previous reports [12] and authors’ clinical preference.

Equine recombinant IL-1β was used in this experiment to induce a pro-inflammatory environment in cartilage explants in vitro [13, 14]. When only considering the main effect of IL-1β, significant differences were observed in media GAG levels of samples stimulated compared to non-stimulated, on Days 2 and 6. The increase in media GAG is an expected result and can be due to increase in catabolism as a result of IL-1β exposure [15]. In reported studies using the same system, the explant exposure to IL-1β has been throughout the entire study and results in not only increased GAG released into the media but also depletion of the total explant GAG and retained labeled GAG at the termination of the experiment [13, 14]. In the present study, IL-1β stimulation at only two time points did not induce significant catabolism measured in the cartilage explants assessed by total or labeled GAG. From these results the authors concluded the system to be valid but the magnitude not as profound as previously observed with IL-1β continually present in the media [13, 14].

In this study the authors assessed “protection” of PL treatment to cartilage explants in a pro-inflammatory environment. The results did not demonstrate statistically different or protective effects of PL treatment when compared to ITS+IL-1β or FBS+ IL-1β controls for any outcome parameters, the latter finding was surprising to the authors. Previous studies that have reported both anti-catabolic and anabolic effects of PRP in other in vitro culture systems [16, 17], had different methods of inducing a pro-inflammatory environment [16] and study design [17] compared to the present study. For this reason, we believe that the differences observed between our findings and other studies could be due to differences in the inflammatory stimulation and systems used [16, 17].

While the application of IL-1β to induce catabolic condition in cartilage has been used extensively in research [11, 16], this in vitro system does not consider the inter-cellular communication that occurs between tissues and the inflammatory progression that naturally occur over time within OA joints [18]. In addition, it does not allow for evaluation of the clinical beneficial effects of PRP, observed previously in in vivo studies [5, 19]. Consequently the effects of the proposed PL formulations and the potential disease modulatory effects and clinical improvement as observed in previous studies using PRP derived products in vivo [4, 5, 19] could not be fully assessed in this in vitro system.

Although freeze/thaw has been used extensively [20, 21] as a method to induce bioactive factor release [20], it is important to consider that differences in PRP preparation and activation (such as freeze/thawing) could have influenced our results when compared to other studies [20]. Therefore the use of exogenous activated or non-activated fresh PRP [20] in place of frozen PL could potentially have resulted in different protective effects in this in vitro system. Thus, a detailed study comparing the effects of allogeneic freeze-dried and autologous fresh platelet-derived product (exogenously activated and non-activated) is important for further evaluation of this product before performing studies in vivo. Further optimization of the PL used in this study may be necessary for more significant protective effects.

No significant Treatment effects were noted in any of the outcome parameters. These results agree with other studies that compared the use of freeze-dried and frozen PRP in wound healing [22]. Specifically, freeze-dried platelet preparations demonstrated similar increase in granulation, proliferation and angiogenic response compared to frozen PRP in a diabetic wound mouse system [22]. Thus, we did not find any evidence to reject equivalency between the frozen compared to the freeze-dried PL.

When focused on the effect of Allogenicity on PL no evidence of non-equivalency between allogenic or autologous PL was observed. Supporting this conclusion, a previous report comparing autologous and allogeneic forms of PRP or PPP in fibroblasts in vitro, concluded autologous and allogeneic forms were equivalent for cell migration and proliferation [23]. Moreover, in a large bone defect in vivo system, allogeneic PRP demonstrated to be efficient in enhancing bone healing providing more consistent quality repair compared to autologous preparations [24]. Taking into consideration our findings in addition to previous studies [23, 24], we believe the allogeneic PL tested demonstrated equivalency to the autologous version of this product.

When the controls were not taken into consideration for comparison, the main effect of Concentration did, however, demonstrate a significant effect in the current system. Specifically, the 3-fold PL concentration had significantly more ^35^SO_4_-labeled GAG retained in the cartilage explants at study termination when compared to both 1.5 and 6-fold concentrations. When taking the controls into consideration for the comparison, samples treated with 3-fold PL still presented higher ^35^SO_4_-labeled GAG concentration (142.85 DPM/μg of DNA) compared to controls (34.85 DPM/μg of DNA) however, not statistically significant (P:0.078). The lack of statistical significance when taking into consideration the controls may be explained by the high variability in ^35^SO_4_-labeled GAG values among the horses used in the study. A greater number of subjects in a future study may lead to a significant result.

Still, the increased concentration in ^35^SO_4_-labeled GAG in cartilage in both comparisons can be interpret as an indicator of less degradation at the 3-fold concentration compared to 1.5 and 6-fold given equivalent total GAG in the cartilage. This is in accordance with previous reports that suggested that higher concentrations of platelets within PRP did not result in better effects in cartilage [11, 25]. Kisiday et al. (2012) [11] showed similar cartilage matrix synthesis with the use of three different concentrations of double spin PRP (3x, 6x and 9x) in cartilage explants stimulated and not stimulated with IL-1β. The authors concluded that actually a single-spin PRP demonstrated to be potentially more advantageous to be used in joints, compared to double spin protocols resulting in higher platelets concentration [11].

Another reported effect has been that excessive platelet concentrations can lead to inhibitory effects [25, 26], which might be due to a concentration-dependent negative feedback. An excessive number (> 1,000,000 cells/μl) of platelets can lead to apoptosis, growth factor receptor downregulation and receptor desensitization resulting in a paradoxical inhibitory effect [26]. While highly concentrated PRP might lead to inhibitory effects [25, 26], low concentration of platelets may lead to insufficient stimulation, as demonstrated previously in a rabbit system of peri-implant bone regeneration [26]. In different studies, an intermediate concentration of platelets seems to present optimal results [25, 26].Moreover, it was demonstrated that the growth factor concentration in PRP is not necessarily correlated with the number of platelets [27]. While it appears consensus has not been reached in the literature on the effect of platelets concentration, based in our model and previous studies [25, 26] an intermediate concentration of platelets in PRP should be considered for joint tissue.

In this study, a few potential pitfalls should be recognized. The authors did not evaluate the growth factors present within the PL preparations or in the culture media in this experiment. The authors are aware that the different preparations of the PL may influence in its growth factor content. However, this experiment is the first step for the development of a potential commercial product, and the main objective was to evaluate if allogeneic freeze-dried would have similar biologic effects compared to autologous frozen PL in an in vitro system. Once the authors observed similar effects between groups, it was assumed that the concentration of cytokines (and growth factors) were equivalent, or that the differences in modulatory effects would not be relevant enough to be reflected in the cartilage explants treated under inflammatory condition. Yet, further detailed investigation of the different preparations is recommended for better understanding and future applications in vivo.

Direct assessment of the platelet and nucleated cell count of the different PL tested in this experiment was not performed due to platelet lyse after thawing the pellets. As mentioned previously, platelets were frozen to preserve their properties, as a form of activation and due to logistical concerns. Still, estimation of the platelet and nucleated cell count was possible considering the counts of the baseline platelet product, allowing the authors to test the effects of different concentrations of PL.

Histopathology evaluation was not performed in this study. Although this can be considered as a potential pitfall, the analysis of the total amount of GAG in cartilage as well as the study of its degradation provided the authors with solid base for evaluation of the effects of IL-1β and PL in cartilage. Additionally, the biochemical analysis performed in this study done in place of the histopathologic analysis allowed the authors to evaluate the effects of PL in cartilage over time (through the evaluation of the GAG present in media in comparison with GAG present in cartilage explant). We are aware that the histopathological evaluation of cartilage samples, especially if performed in different time points of the experiment would be ideal for a detailed evaluation of PL effects in cartilage. However, the number of cartilage samples required for such analysis would be much higher and not possible due to the design of the current study.

## Conclusions

In conclusion, the authors observed the system used in this study provided modest inflammatory response in cartilage to test the various treatment groups. With no evidence that the conditions studied were dissimilar (allogeneic freeze-dried versus autologous frozen), the authors believe that allogeneic freeze-dried platelet derived products could potentially be used in place of frozen PL. However, we do emphasize that such assumptions need to be clarified and the protective effects of allogeneic freeze-dried PL reinsured, comparing this product with fresh PRP in future experiments. We did find differences based on concentration that favored the 3-fold PL and based on these findings, intermediate concentrations of platelets in platelet derived products should be considered for use in joints.

## Methods

### Animals

Tissue from eight skeletally mature and healthy horses (2–5 years of age) was used in this in vitro study. The horses used belonged to a terminal experiment not related to the present study. Horses were purchased from a commercial vendor that adhered to all Colorado State University and accrediting guidelines. From these horses, blood was collected for PRP preparation and cartilage was harvested after euthanasia. Horses were sedated with xylazine (1 mg/kg) and euthanized with an overdose of pentobarbital (120 mg/kg) administered intravenously. All the procedures performed for the current experiment such as blood draw and tissue collection, as well as the euthanasia method described adhered to the Colorado State University Animal Care and Use Committee (ACUC) guidelines and were approved (ACUC, protocol number: 12–3879).

### Platelet lysate preparation

Blood was collected and centrifuged in our lab to produce the PRP as described [11]. Briefly, blood was collected from the horses into 450 mL blood bags containing 63 mL of anti-coagulant citrate phosphate dextrose adenine (CPDA). The blood was then placed in 50 ml conical tubes and centrifuged at 200 g for 18 min. The supernatant above the buffy coat with the platelets and plasma was collected, creating a baseline platelet product. The baseline platelet product was centrifuged again for 10 min at 1000 g to pellet the platelets. A small aliquot of the baseline platelet product was used for manual and automatic platelet counting.^4^ The supernatant (platelet-poor plasma) was collected and all platelet pellets were frozen at − 80 °C. Although differences have been observed in fresh versus frozen PRP [28], due to logistical concerns and because freezing has been demonstrated to be a valid method to induce the release of growth factors [20], frozen PL was used in this experiment in place of the fresh PRP used in the field.

The platelet pellets were divided into three groups of treatments. In one group, the pellets were lyophilized for minimum of 18 h (Freeze-dried PL) and kept at − 80 °C until the commencement of experiment, while the Frozen PL group remained stored at − 80 °C. The Filtered freeze-dried PL group was processed similarly as described for the freeze-dried PL however, to remove the platelet debris the product was filtered using a 33 mm syringe low biding filter^5^ prior to lyophilization. The Frozen and Freeze-dried groups were compared to address the objective of the study. The filtered freeze-dried group had the purpose of evaluating the influence of the platelets (or it’s debris) in platelet lysate’s effect. For reporting purposes, we will refer to the different PL formulations (Frozen, Freeze-dried and Filtered freeze-dried) collectively as Treatment.

**Fig. 3:**
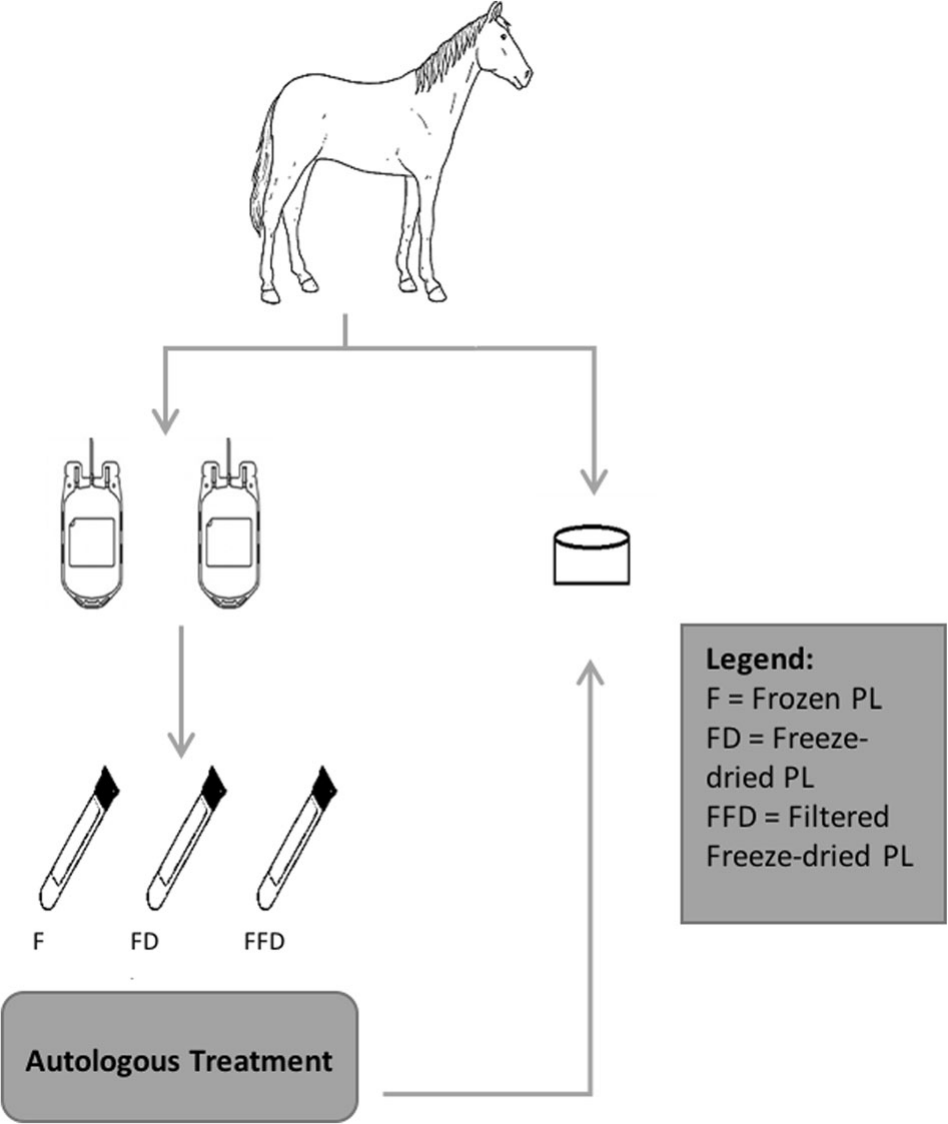
Autologous PL treatment used in the experiment. Blood was collected from 8 horses and used to process the PL. The PL was applied into the cartilage of the same horse the blood was collected

**Fig. 4:**
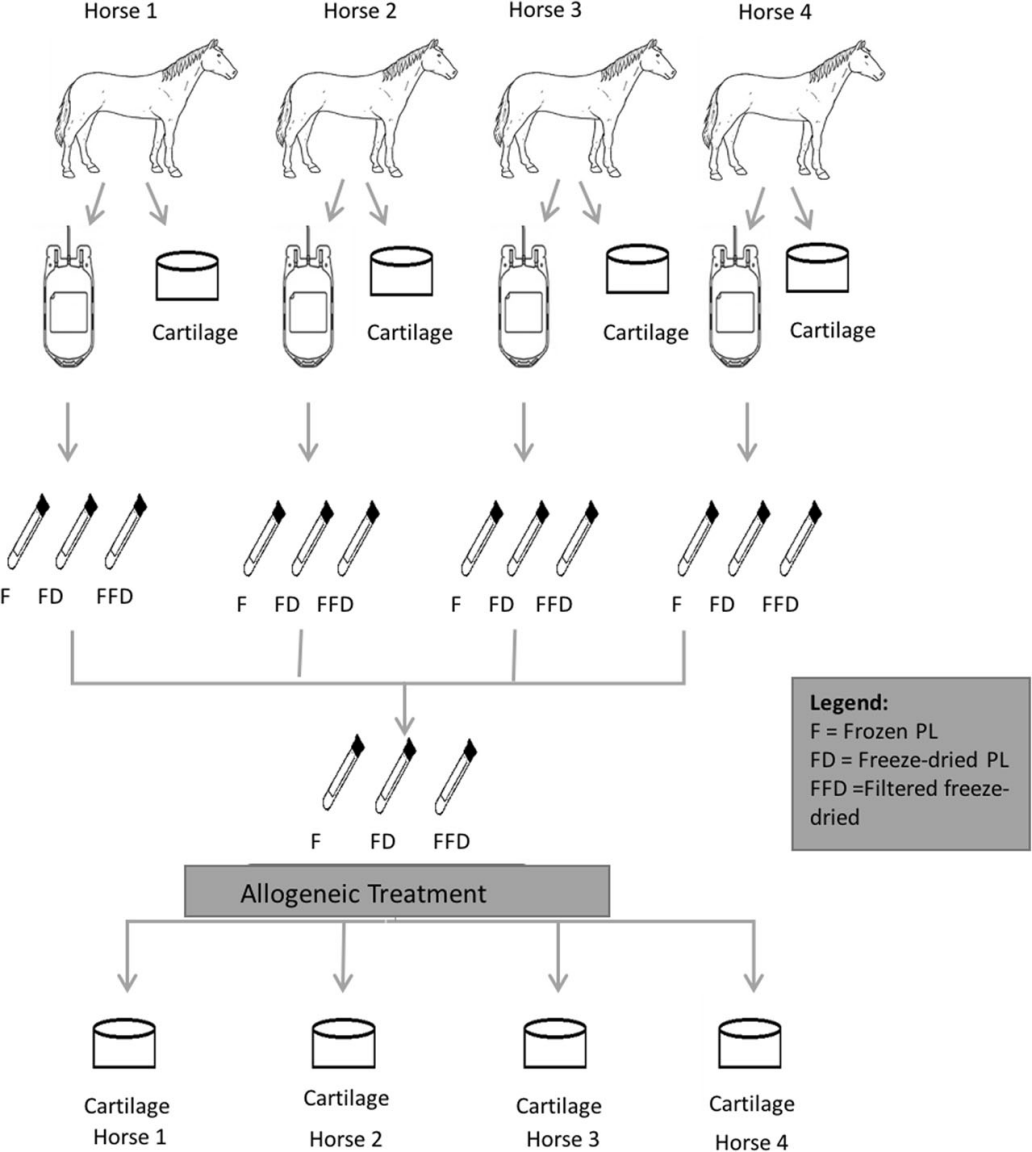
Allogeneic PL treatment. Blood was collected from different horses to produce the PL. The PL produced was combined and applied to cartilage from different horses

We also assessed Treatment based on autologous and allogeneic source of material. For the autologous PL treatment, the platelet pellets were diluted in media [Dulbecco’s Modified Eagles’ Medium – DMEM^6^ supplemented with 1% volume/volume of insulin transferrin selenium (ITS Premix^7^); 1 mM nonessential amino acids, 10 mM HEPES, 0.4 mM proline, 0.11 mM ascorbic acid, penicillin (100 U/mL) and streptomycin (100 μg/mL)] and applied to cartilage from the same horses the blood was collected (Fig. 3). For allogeneic PL, a combination of different horse material was utilized and applied in cartilage cultures of different horses in two groups of *N* = 4 (Fig. 4). For reporting purposes, we will refer to the condition of autologous or allogeneic material collectively as Allogenicity.

We tested the Treatment and Allogenicity at three different concentrations. Based on the average platelet concentration in whole blood from the study population platelet pellets from each horse were re-suspended in media in different volumes to create Treatment concentrations of 1.5, 3 and 6-fold the baseline platelet count, in both allogeneic and autologous materials. For reporting purposes, these different concentrations will be collectively referred as Concentration.

### Cartilage explant harvest

Immediately after euthanasia, cartilage from trochlear ridges and condyles from the stifle (knee) joint of each horse was harvested using an 8 mm punch (wet weights between 70 and 100 mg). Explants were placed in a 24 well plate that contained DMEM media (same media used for PL preparation).

### Treatment preparation & cartilage culture

PL pellets representing Frozen, Freeze-dried and Filtered Freeze-dried were thawed and diluted in ITS media based on intended final platelet concentration as well as considering autologous or allogeneic conditions when explants were added. All permutations were run in duplicate. Because unquantified growth factors in routine fetal bovine serum (FBS) media could potentially interfere with the effects of the PL, a 1% ITS supplemented media [29, 30] was chosen for this experiment. Control explants were exposed to base media containing ITS or 10% FBS (to allow comparison to other studies). All samples were then allowed to equilibrate in the designated medium for 48 h in humidified incubator at 37 °C before Treatment application.

**Fig. 5:**
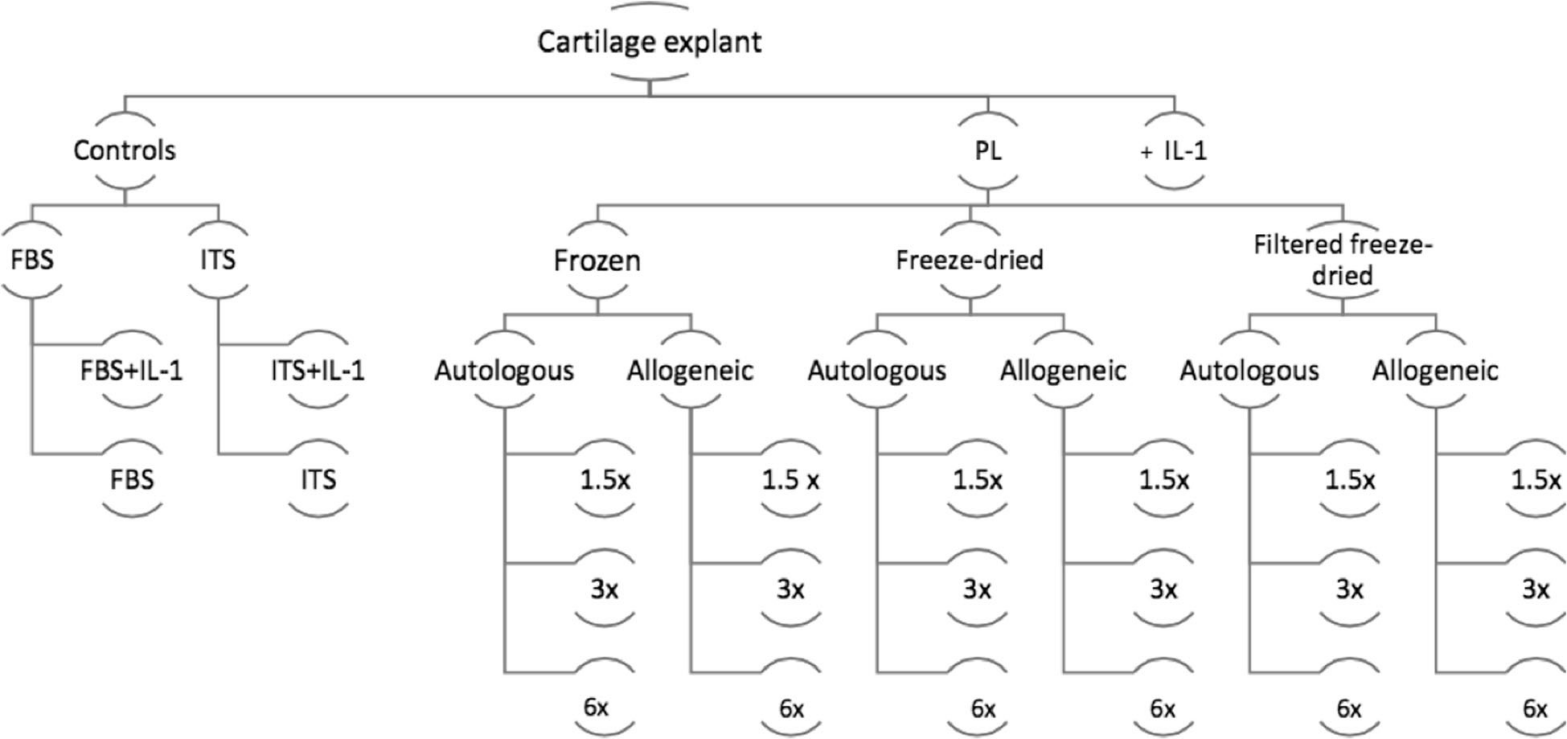
Diagram of PL groups and controls used in this study

**Fig. 6:**
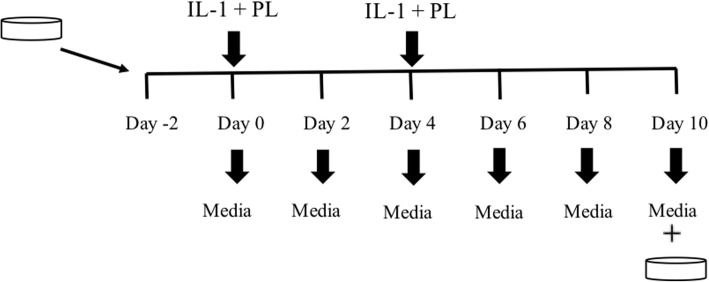
Timeline of the study. Cartilage samples were collected and let to equilibrate in media for 48 h before the commencement of the experiment. Then, at day 0 and 4 samples were exposed to IL-1β and PL. All the other days of the experiment explants received full growth media only. Media was collected every-2 days and at the end of the study, cartilage and media samples were collected

The Treatment was applied twice throughout the study on day 0 and day 4, IL-1ß was also added to the media on these days to expose explants to an inflammatory environment [IL-1ß^8^ (10 ng/mL) [11, 13] diluted in 0.1% bovine serum albumin^9^ (BSA) and PBS]. Stimulated controls were exposed to IL-1β on days 0 and 4 as well. Non-stimulated controls (cartilage in plain medium) were left untreated. Media was replaced every 48 h and stored at − 80 °C. On the 10th day of the experiment all the cartilage explants as well as the media were collected and frozen at − 80 °C until analysis (Figs. 5 and 6).

### Biochemical analysis

At the termination of the experiment cartilage explants were harvested and then lyophilized followed by overnight papain digestion at 60 °C using a crystallized papain suspension [ 31].

#### Explant DNA quantification

Cartilage explant DNA content was determined using a fluorescent dye-based assay (Hoechst 33258^10^) [32]. Samples were run in duplicates and were read against a standard curve using calf thymus DNA. DNA content was normalized to explant dry weight (μg of DNA/mg of cartilage dry weight).

#### Explant and media glycosaminoglycan quantification

Cartilage explants and media were analyzed for total GAG content using a modified method of the dimethyl methylene blue assay [33]. Samples were run in duplicate and compared to a standard curve using chondroitin sulfate C. GAG was normalized to DNA content in cartilage and presented as GAG in μg/μg of DNA.

#### Explant ^35^SO_4_-labeled proteoglycans quantification

Newly synthesized GAG was labeled by the addition of 5 μCi of ^35^SO_4_ sixteen hours prior to Day 0 to the media of all cartilage explants. Following the experiment, the ^35^SO_4_ within cartilage explants was quantified using a modified scintillation count method with Alcian blue dye as detection system [34]. Samples were run in duplicate and compared to a standard curve containing different concentrations of ^35^SO_4_ [34]. The level of activity of the ^35^SO_4_ bound to the GAG molecules were estimated in disintegrations per minute (DPM). Data was normalized to DNA content and presented as DPM/μg of DNA. These data were used as a measure of GAG retention or indirect measure of degradation of newly synthetized GAG.

### Data analysis

Analyses were performed using a mixed-model analysis of variance (PROC GLIMMIX, SAS version 9.3^11^) [35]. First, we evaluated the effectiveness of the model evaluating the main effect of IL-1β stimulation on the explants. Then, we evaluted the protective effects of PL treated samples compared to IL-1β stimulated controls and finally we considered the fixed effects of Treatment, Allogenicity and Concentration as well as all interactions between these effects in samples exposed to IL-1β. Media GAG, explant GAG and ^35^SO_4_-labeled GAG were considered as dependent variables for all three analyses. Student residual plots were used to ensure normality and log transformation was performed as indicated. Restricted Maximum Likelihood was used as estimation technique in this experiment. Protection against multiple comparisons was achieved by using a protected F test. Individual comparisons supported by the F-test were done using least-squares means procedure. In all statistical comparisons, *P* value < 0.05 was considered significant.

## Data Availability

The datasets used and/or analyzed during the current study are available from the corresponding author on reasonable request.

## Abbreviations

CPDA: citrate phosphate dextrose adenine
DMEM: Dulbecco’s modified Eagle’s medium
DPM: Disintegrations per minute
FBS: Fetal bovine serum
GAG: Glycosaminoglycan
IL-1β: Interleukin-1β
ITS: Insulin transferrin selenium
OA: Osteoarthritis
PBS: Phosphate Buffered Saline
PDGF: Platelet derived growth factor
PL: Platelet-lysate
PRP: Platelet-rich plasma
TGF-β: Transforming growth factor beta
μCi: Micro Curies

## Footnotes

1 Values defined by normalization studies performed at the Veterinary Diagnostic Laboratory, Colorado State University

2 Values defined by normalization studies performed at the Veterinary Diagnostic Laboratory, Colorado State University

3 Values defined by normalization studies performed at the Veterinary Diagnostic Laboratory, Colorado State University

4 Siemens Advia 120 Automated Hematology analyzer

5 33 mm syringe low binding filter with PES membrane (45 μm) MilliporeSigma, Burlington-MA

6 DMEM GIBCO, Thermo Fisher Scientific, Waltham, MA

7 ITS Premix 6.25 mg of insulin, 6.25 mg of transferrin and 6.25 μg of selenium acid -BD Bioscience, San Diego-CA

8 IL-1β, R&D Systems, Minneapolis-MN

9 BSA, Sigma Chemicals, Saint Louis-MO

10 Hoechst 33258 Sigma Chemicals, Saint Louis-MO, U.S.A

11 PROC GLIMMIX, SA, version 9.3, SAS Institute Inc., Cary, NC
